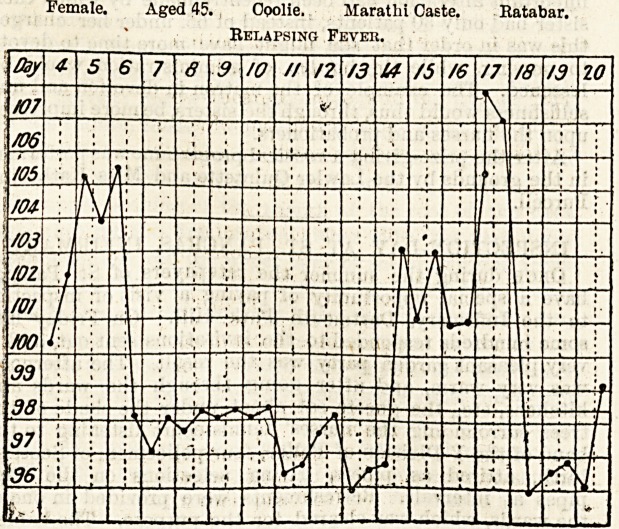# "The Hospital" Nursing Mirror

**Published:** 1901-07-13

**Authors:** 


"The Hospital" Nursing Mirror.

Being the Nursing Section of "The Hospital."

[Contributions for this Section of "The Hospital" should be addressed to the Editor, "The Hospital" Nursing Mirror, 28 & 29 Southampton Street Strand, London, W.C.]

### Notes on News from the Nursing World.

### RECEPTION OF PENSION FUND NURSES AT MARLBOROUGH HOUSE.

Our readers will learn with much interest and pleasure that her Majesty the Queen has graciously intimated her intention of presenting certificates to the eighth and ninth thousand nurses who have joined the Royal National Pension Fund for Nurses, at Marlborough House, on Friday, the 19th instant. This, the most important nursing event of the year, will be the fifth occasion on which the Pension Fund nurses have been received at Marlborough House, the last being on July 21st, 1899; but in one sense the function will be unique, as the forthcoming reception is by the Queen Consort, instead of by the Princess of Wales. The nurses invited by her Majesty, the President, are the policyholders whose policies are numbered from 7,251 to 8,700, both inclusive the existing members of the Fund, who, though entitled to receive certificates from the President at previous receptions, have been unable to attend, and all members of the Fund who have been on active service at the war, or on plague duty. The reception will, therefore, be of a most comprehensive character, and will, as her Majesty clearly intends, afford the maximum of gratification to her nurses. A preliminary meeting will be held in the grounds at Marlborough House on the morning of the day, which every nurse who wishes to be at the presentation must attend. Uniform will, of course, be essential, and each nurse will wear the charming armlet of the Pension Fund. For the guidance and information of members, the secretary asks us to state that cards containing the fullest detailed instructions should be received not later than Friday this week, and it will obviously save an immense amount of trouble if they do not communicate with the office before they are in possession of their cards. Matrons and superintendents of Homes for Nurses, or others who are able to put up nurses on the evening of Thursday, the 18th, will greatly oblige by at once informing the secretary the nature of the accommodation they have at their disposal, stating the terms for bed and breakfast. The office of the Fund, at 28 Finsbury Pavement, E.C., will be closed on the day of the ceremony.

### THE NEW "QUEEN'S" NURSES.

The full list of new "Queen's" Nurses, as approved by Her Majesty Queen Alexandra, appears in another column. It will be observed that six of them are working in Liverpool, four in Birmingham, three in London, three in Gloucester, ten in Edinburgh, and six in Dublin. The Scottish and Irish contingent were all trained in Scotland and Ireland, and a fair proportion of the English at the Central Home in Bloomsbury. Lady Hermione Blackwood is working at Gloucester.

### A CRY FROM HONG KONG.

It is not only in Cape Colony and British Columbia that more nurses are wanted. A cry of distress reaches us from the colony of Hong Kong, in which the plague continues to rage. According to a British resident, one of whose friends unfortunately contracted the d s as , Europeans are "bundled out of their homes, at the mercy of Chinese coolies, who bang them down in the street, and sit and smoke. When the Civil Hospital is reached there is no one to attend to them for hours." Obviously, there should be a nurse, as well as a doctor, ready to receive the plague patients, and her absence under such circumstances can only be attributed to grave negligence or to an inadequate nursing staff. In the latter case the increase in the supply should, of course, be made by the representatives of the British Government. It is as important that sufferers from plague in Hong Kong should be properly nursed as it is in South Africa, and the question calls for the immediate attention of the Colonial Office at home. This is the more urgent as about eighteen Europeans altogether have been attacked, and there have been several deaths. Of three young fellows in the Hong Kong dispensary, two are among the victims.

### THE QUESTION OF NEGLIGENT NURSES.

An action brought against the Massachusetts Homœopathic Hospital by a patient to recover damages for an injury alleged to have been sustained by the negligence of a nurse in the hospital, who, it was alleged, placed a hot-water bag close to the plaintiff's side, thereby burning her unusually sensitive skin, has been finally disposed of by the American Court of Appeal. When it was first tried in the Circuit Court, the counsel for the plaintiff based his case on the fact that his client was a paying patient, and contended that the care of patients who pay was different from that of those who are treated gratuitously. The Circuit Court declined to accept this plea, and the Court of Appeal, in confirming the judgment, held that the degree of protection from unskilled and careless nurses must, "in a hospital maintained for charity," be the same for paying and for non-paying patients. That is to say, it ruled that a patient admitted to a hospital maintained for charity cannot, just because payment has been made, recover damages for injuries caused by negligence. It would obviously be wrong that rich patients who are allowed, on payment, to have the privilege of commanding the treatment available at a hospital should be placed in a position to divert funds obtained for a charitable purpose into their own pockets for any reason whatever. But a hospital, either in America or in this country, would soon cease to receive public support if it were believed that the patients were not skilfully nursed. The known tolerance of negligent nurses would mean the withdrawal of subscriptions.

### OUR BELGIAN NURSE.

Mme. Alice Bron, who is now engaged as a nursing sister on night duty at No. 10 Stationary Hospital, Naauwpoort, and finds her time fully occupied, will not be greatly disturbed by the savage attack which has been made upon her in a Belgian paper. She may not even think it necessary to take any notice of a censor who asserts that she and her companions had to "run away for their lives from their ambulance, which was, despite its having the Red Cross flag, being bombarded by English troops," but omits to notice her remark that "the English doubtless saw our white caps and aprons through their telescopes, and took care to spare the sisters, for whom they have such a great respect."

### NURSING BY RELIGIOUS ORDERS.

A correspondent who signs herself "Fair Play" strangely misunderstands some remarks which we have made in reference to nursing by members of religious orders. There has been nothing in the nature of an attack on these estimable ladies, who, personally, are entitled to be held in the highest esteem, and though we quoted the assertion of a doctor at Lille that the hospitals in that city were neglected "owing to so much time being spent upon religious observances," we did not endorse it. The reasons of our preference for lay nurses have been stated so often that they need not be repeated, and it should hardly require to be pointed out that a fully-trained lay nurse may be as deeply religious as a nun.

### LECTURES TO NURSES OF THE LONDON HOSPITAL.

The "talk" to the nurses of the London Hospital by Mr. Sydney Holland, the chairman, in 1897, which was subsequently published as a couple of lectures, evidently appeals to a wider circle. The pamphlet has now, we are informed, reached the seventh thousand. One reader was so pleased with it that he bought 800 copies from the publishers and presented it to each of the "Queen's" nurses. Several hundred copies have also, it is stated, been ordered and sent for distribution in the Transvaal and Natal.

### WORKHOUSE NURSING IN YORKSHIRE.

Last week a conference, largely attended, was held at the Union Offices, Leeds, in order to discuss the training and certificates of workhouse nursing in Yorkshire, and to consider a report that had been prepared by a special committee which was appointed by a similar conference at the beginning of the present year. It will be remembered that on that occasion the feeling was general that there should be a uniform standard of training and examination for nurses in workhouse infirmaries, and a committee was chosen in order to draw up a workable scheme for the examination of candidates for the position of certificated nurse, and for giving them certificates. The conference had before them the scheme of the committee, whose recommendations were as follows:—

> That a board be formed from the contracting Unions to be known as the Yorkshire Poor Law Nursing Board; that applicants for the position of probationer nurse should serve a period of at least two months on trial, and if found suitable they should enter into an agreement with the Union engaging to train for a period of at least three years; that the training to be given in all the contracting Unions be assimilated as far as possible, regard being paid in providing for this to conditions prevailing in the smaller workhouse infirmaries; and that a supervising training committee, consisting of five lady superintendent nurses, be formed to give advice and assistance on matters of training to those Unions seeking it—such committee to have charge of the examination of probationers from all the contracting Unions in their practical nursing work.

In the course of an interesting discussion, it transpired that the Yorkshire College had expressed its willingness to assist in that part of the scheme which consists in testing the theoretical knowledge of those nurses where practical efficiency is satisfactorily guaranteed by the Poor Law Unions. The College proposed that in the case of all nurses presented for examination to the medical department it should be understood that the practical training which they have received has been given at least for a certain minimum length of time in hospitals which reach a certain standard as to the number of beds, variety of cases, and methods of instruction. This proposal the committee had approved, and the conference cordially endorsed it. Subsequently, it was agreed to form a Yorkshire Poor Law Nursing Board consisting of one representative from each contracting union, with power to co-opt five medical officers and five superintendent nurses as members, the Yorkshire College to be also represented on the board. The unions joining the scheme will be asked to bind themselves by agreement for a period of three years to carry it into effect.

### A SHABBY CORPORATION.

A striking instance of the manner in which public bodies should not treat their employés was given last week in Halifax County Court. Miss Sarah Ann Moore, a hospital nurse, was plaintiff in an action against the Halifax Corporation. Her case was that in May, 1900, she was engaged by the Corporation to take charge of the washing section of nursing duties at a temporary smallpox hospital at Luddenden. Her wages were 24s. per week and board and lodging. After being on duty three weeks the nurse was stricken down with smallpox, and she had herself to be nursed for upwards of two months. The Corporation offered her three weeks' wages, which she declined to accept, and claimed for the time she was discharged from hospital, namely, thirteen weeks. The validity of the claim was recognised by the judge, who ordered the Halifax Corporation to pay the full amount. We cannot understand how it came to be disputed. That a nurse who in the discharge of her duty contracts a disease should be penalised by depriving her of salary during the period of her illness, is a monstrous doctrine. If it were allowed to obtain, nurses would be justified in declining to tend patients suffering from infectious disease. It is their duty to run the risk of contagion, but it is the duty of those who employ them to see that they do not suffer in pocket as well as in health.

### THE NURSES AT ST. PANCRAS INFIRMARY.

The Nurses' Home at St. Pancras Infirmary has recently been thoroughly cleaned and re-decorated, and the nurses have not long been back in their own quarters. While the rooms were in the hands of the workpeople they occupied one of the wards, which was set apart for the purpose. The matron speaks most highly of the helpful way in which the staff gave all the assistance in their power towards "getting things straight." When the home was again ready for occupation a great deal of extra labour was involved, and the nurses, she says, devoted time and energy ungrudgingly during a necessarily somewhat trying period. Their large and airy "common room" now looks most inviting, with its comfortable chairs and sofas, its piano and flowers. The four large windows give plenty of light, even when the room is divided so as to make two, in one of which lectures are given. The dining-room is another cheerful apartment, and all the rooms, upstairs and down, are coloured in terra-cotta. The senior nurses have separate bedrooms, and the sisters and night nurses have their own sittingrooms, all nicely furnished and very cosy-looking. The infirmary stands high—indeed, a sundial in Waterlow Park, which is close by, is said to be the same height as St. Paul's Cathedral—consequently the view from the windows is an exceptionally fine one, taking in the Tower Bridge, the Great Wheel at Earl's Court Exhibition, and even, on clear days, the Crystal Palace.

### AN INCIDENT OF "SPRING CLEANING."

A NURSE reports an amusing incident which occurred a few days ago prior to an operation in "private nursing." Having arrived at the house within an hour or two of the time appointed for the operation, she was rendering the room as antiseptic as possible under the circumstances, and was in the act of spreading a clean sheet over the dusty-looking carpet, when she was beckoned out by the mistress of the house and informed "that she needn't be so particular about the room, as it was very dirty, and was the only chamber in the house which had not been 'spring cleaned'—and had been chosen for that reason," the date being the end of June. It can be understood with what difficulty the nurse kept her countenance under control while this information was being solemnly imparted to her. She adds that she is quite sure that, though the incident happened, not in a cottage, but in a young ladies' boarding school, the surgeons in requiring these preliminary arrangements were credited with having only the kindest consideration for the furniture in the room.

### IRISH GUARDIANS AND UNTRAINED NURSES.

AT the monthly meeting of the committee of management of Clare County Infirmary a letter was read from Miss Annie Minogue, of the Tulla Union, asking that she might be allowed to undergo a course of training as probationer nurse, so that she might be qualified, as trained nurse, to meet the requirements of the Irish Local Government Board. One of the Tulla Guardians stated that Miss Minogue had been appointed nurse by their Board, but as she was not a "trained nurse" the L.G.B. refused to sanction her appointment. He also said that one of the Board's inspectors had since stated that "if the girl put in a year at an institution like the Clare Infirmary the Board would then favourably consider her appointment." Ultimately it was arranged by the committee that Miss Minogue should be taken for a premium of £10, payable in quarterly instalments. But nothing was arranged respecting the nursing at Tulla Union in the meantime. We suppose that another "untrained nurse" will be temporarily appointed until the probationer has completed her year's training. It is more satisfactory to observe that, on the suggestion of a member of the managers of Clare Infirmary, an appeal has been addressed to the Local Government Board, requesting that in framing new rules for nursing in the several workhouses the Board will draw them up so as to include county infirmaries with a duly qualified surgeon and matron —as at Clare—among the training institutions for workhouse nurses. But the term of training should be extended.

### A NURSES' HOME FOR CHESTER INFIRMARY.

IT has been decided by the governors of Chester Infirmary, at the instance of the Board of Management, to spend a sum of £1,300 on the conversion into separate dormitories of the detached wing of the hospital formerly appropriated to the treatment of infectious cases, and its proper equipment as a nurses' home. Until now, two houses at the rear of the infirmary have been made to answer the purpose, but the accommodation afforded has for a long time been found insufficient and unsuitable. In asking the governors to sanction the expenditure, the chairman, while not admitting that a justification was necessary, mentioned that the nurses had given material assistance to the institution, the clear gain on their services since 1892 having been £2,500, or nearly double the amount which it is intended to spend in order to make them more comfortable.

### MUNICIPAL NURSES.

A FEW weeks ago Dr. Richards, the Medical Officer of Health for Croydon, presented his first annual report, in which he pointed out that the death-rate amongst infants was very excessive, amounting to 132 per 1,000, and proceeded to say: "It would be no exaggeration to assert that at least one-half of the deaths were preventable, more especially those that can fairly be assigned to dietic errors. The latter include most of the deaths from diarrhœa, enteritis, debility, and convulsions." On Monday the Croydon Council further considered the matter, and on the recommendation of the Sanitary Committee agreed to the following suggestions presented by Dr. Richards.

In reference to the questions raised in my annual report as to infantile mortality and school hygiene, I suggest that arrangement be made whereby the present borough nurse is relieved of all responsibility in connection with investigation of notifiable diseases.

That a second nurse or health visitor be appointed at the same rate of salary, namely, £95, rising to £100, with uniform.

(a) That it be the duty of these two women inspectors to visit from house to house in those districts where the medical officer directs them to do so.

(b) They would be supplied with a list of births occurring in the poorer parts of the town, and would be required to visit the homes and give practical instruction in infant feeding and management.

(c) They would make note of the general sanitary condition of the houses visited, direct the attention of the tenants to the evils arising from the want of domestic cleanliness, and report to the Medical Officer of Health any structural defects.

(d) They would instruct the parents of children as to the precautions to be taken in the case of measles, whooping-cough, and other children's complaints reported to us by the school authorities.

### SHORT ITEMS.

The s.s. *Victorian* arrived last week with Sisters M. A. Hartwell and H. A. Legg on duty; both return to South Africa.—The s.s. *Montford*, arriving on Friday, brought Sisters N. G. Hill, J. A. Clarke, W. A. Naylor, and J. M. Lempriere (leave).—The s.s. *Dilwara* arrived from Malta on Sunday, with Sisters Wright and Bridgford (on leave). —The *Dunera* and the *Roslin Castle* came in on Monday from South Africa; Sisters C. M. Mills, K. Moron, L. Sands, H. Millar, E. Andrews and H. Kinealy are on leave, and will rejoin the *Dunera*; the *Roslin Castle* had on board Sisters F. E. Cross and D. Burgess (Colonial Nursing Service).—Sister M. E. Howell, of the Army Nursing Service Reserve, is reported, under date of July 4, "dangerously ill" at Aliwal North.—We are informed that the Sheffield Royal Infirmary is now a three-years training school, and that no certificate is granted for less than that period of training.—On Monday afternoon an entertainment was given at 29 Berkeley Square, in aid of the Affiliated Benefit Nursing Associations, an organisation of certificated monthly nurses with general training.

### Notes of Surgical Lectures to Nurses.

Delivered at the London Temperance Hospital by Dr. W. J. COLLINS, M.S., B.Sc., D.P.H.Lond., Fellow of London University and of the Royal College of Surgeons. Surgeon to the London Temperance and the Royal Eye Hospitals.

### THE HEAD AND NECK.

THE neck viewed laterally is divided into two triangles by the great muscle arising from the sternum and clavicle and inserted into the prominent nipple-like process behind the ear called the mastoid (sterno-mastoid); the action of this muscle is to approximate the ear to the shoulder of the same side and direct the chin towards the opposite shoulder. In wry-neck (torticollis) its origin may require division (tenotomy). The external jugular vein may be seen under the skin running from the angle of the jaw over the sterno-mastoid to the middle of the clavicle. The triangle in front of the sterno-mastoid with its base upwards is called the carotid triangle; the one behind it with its base downwards is called the posterior triangle. The carotid artery (with its bifurcation and branches therefrom) in a sheath, which also contains the internal jugular vein and pneumogastric nerve, lies in the anterior or carotid triangle under cover of the edge of the sterno-mastoid muscle; the subclavian artery and vein and their branches and the brachial plexus of nerves lie deeply in the posterior triangle under cover of the clavicle, while a string of glands (concatenate), often enlarged in tubercular disease, follow the posterior margin of the sterno-mastoid.

If the middle line of the neck be traced with the eye and finger, there are encountered from above downward the symphysis of the lower jaw, the hyoid bone, the thyroid cartilage (or Adam's apple), with the lobes of the thyroid gland on either side, the crico-thyroid membrane (through which laryngotomy is performed), the cricoid cartilage and the rings of the trachea, the second and third of which are usually overlaid by the isthmus of the thyroid gland. This ductless gland, enlarged in goitre and Graves' disease and atrophied in cretinism and myxœdema, appears to subserve some function in elaborating the blood; the abolition of it occasions a degeneration of body and mind producing a solid œdema of the skin, altered voice and features, and mental dulness. These symptoms disappear on feeding with sheep's thyroid.

By means of the laryngoscope, the interior of the larynx and trachea may be seen. The epiglottis, made of elastic cartilage, forms a lid to the larynx in the act of swallowing. A gap of varying size separates the mobile vocal cords; these can be rendered tense, slackened, opened, and shut by four pairs of muscles supplied by branches of the pneumogastric nerves; one of these (recurrent) on the left side runs into the thorax, round the aorta, and back to the larynx, a long aberrant course which renders it liable to pressure and consequent paralysis of the left vocal cord.

The upper opening of the thorax at the root of the neck is bounded by the first rib on either side, the first dorsal vertebra behind, and the upper bone of the sternum in front; between the two latter is a space of about two inches. Through this narrow opening pass the trachea, the œsophagus, and thoracic duct; the innominate artery on the right side, the common carotid and subclavian arteries on the left, and the right and left innominate veins converging to their junction in the superior vena cava; the pneumogastric, phrenic, and left recurrent laryngeal nerves; the apices of the two lungs covered by the pleuræ, and several muscles and smaller vessels and nerves. Wounds of the neck bleed freely; wounds of large veins may result in entrance of air and alarming collapse; septic wounds may result in cellulitis, requiring early careful incision. Fatty tumours are common at the back of the neck. Enlarged glands from tubercle, cancer of tongue or breast, pediculi on scalp, carious teeth, or lymphadenoma are common in the neck. The cervical spine consists of seven vertebræ; the first, called the "atlas," carries the globe of the head, and between it and the occipital bone nodding movements take place; the second or axis is so articulated with the atlas as to allow of rotation between them. These movements may be interfered with, and the neck held stiffly in disease (caries) of the cervical spine which may result in abscess pointing behind the pharynx. Fracture or dislocation of spine above the origin of the phrenic nerves (third and fourth cervical) is suddenly or rapidly fatal (as in hanging). The gullet (œsophagus) is 9 inches long, commences at the level of the cricoid cartilage and fifth or sixth cervical vertebra (narrowest part), inclines to left at root of neck (where œsophagotomy may be performed), and ends in the stomach at the level of the ninth dorsal vertebra. Foreign bodies may be removed by the probang; stricture (cancer), not uncommon at either end, may necessitate gastrostomy or opening the stomach to administer food through an artificial mouth below obstruction. Impediment to passage of air through the larynx (causing dyspnœa and dysphonia) may be due to scalds, inflammation, growths, cancer, diphtheria, tubercle, syphilis, foreign bodies, or to paralysis or spasm of the muscles of the larynx.

The cranium consists of eight bones firmly sutured together (occipital, two parietal, frontal, two temporal, sphenoid, and ethmoid), the face of fourteen bones, of which one, the mandible or lower jaw, is articulated by a double hinge joint with the base of the skull immediately in front of the ears.

At birth, and for some months afterwards, soft gaps (fontanelles), two in number, can be felt on the vault of the infant's skull. The anterior is the larger, and does not usually "close" till the fourteenth month, and even later in rickets and hydrocephalus. Between it and the posterior smaller fontanelle lies the sagittal suture, of importance in determining the "positions" in obstetric cases. The thickest part of the skullcap is the occipital protuberance to which the ligamentum nuchæ is attached, the thinnest the roof of the orbit, where punctured wounds are dangerous. The bony prominences above the brows and behind the ears are due to cavities in the bones containing air cells or sinuses; these may be inflamed and may contain pus, requiring to be let out. Neglected discharge of pus from the ear may give rise to mastoid abscess, thrombosis of the great lateral (venous) sinus within the skull and even abscess in the brain or cerebellum.

The cavity of the external ear (meatus) runs first inwards and a little forwards and upwards, and then inwards and a little downwards, owing to an elevation in its floor. This is to be borne in mind in syringing the ear for removal of wax (cerumen) or foreign bodies.

Seven bones form one orbit, and 11 bones form the two orbits, owing to the contribution to the formation of each by the frontal, sphenoid and ethmoid bones. Near the nasal end of the supra-orbital ridge can be felt a notch through which passes one of the many cutaneous nerves which supply the scalp. Near to this spot, but a little further inwards, can be felt the little pulley through which the superior oblique muscle of the eye works in pulling the eye downwards and outwards.

The scalp is richly supplied with blood-vessels, and wounds in this region bleed freely. Blood is sometimes thrown out beneath the scalp, especially in the case of injuries in children, causing a fluid tumour (hæmatoma) and often a deceptive feeling as of depressed fracture at its edge. This is to be distinguished from the "caput succedaneum" on the head of a newly-born infant.

Fractures of the skull may result from violence directly applied or indirectly through the spine or jaw. They may be confined to the base or the vault, or involve the whole skull, and are serious in proportion to the injury inflicted directly or indirectly upon the cranial contents.

The common signs and classification of fractures are in the case of the skull of secondary consideration: the intra-cranial complications (concussion or compression of the brain, meningeal hæmorrhage, injuries to nerves, inflammatory sequelæ) are of predominant importance. Indirect signs of fracture of the base of the skull are: Hæmorrhage from the ears, nose, or mouth, escape of cerebro-spinal fluid, coma, paralysis. Concussion of the brain is the name given to certain symptoms common after severe injuries to the head, even when no fracture may have resulted, and are thought to be due either to bruising of the brain or some disturbance of its blood supply or of the fluid contained in its ventricles. Such symptoms are: Loss of consciousness, collapse, sickness, loss of power over bladder and rectum. In cases of compression of the brain, arising from depressed fracture, hæmorrhage between skull and dura mater or from inflammatory products, there is more profound and increasing coma, embarrassed respiration (Cheyne-Stokes), paralysis (unequal pupils, squint, ptosis, facial palsy, etc., and incontinence of urine and fæces), and a slow or irregular pulse. For the relief of depressed fracture, or for other causes of "compression," for tumour, or to evacuate pus, etc., the cavity of the skull may require to be opened by the chisel, drill, saw, or trephine. Hernia of the brain may ensue upon any deficiency in the skull-cap. In all cases of coma, supreme importance is to be given by the nurse to measures directed to the prevention of bed-sores (cleanliness, water-bed, keeping dry, spirit lotion, zinc ointment, zinc oxide and starch powder). Shaving the scalp and application of cold to the head by india-rubber tubing, through which cold or iced water runs by siphon action, are employed in cases of concussion, and a purgative is usually administered. Fracture of the facial bones and jaws require strict attention to cleanliness in view of proximity to nasal and buccal cavities. Fracture of the lower jaw is usually treated by moulding and applying a gutta-percha splint (rendered supple by hot water) and securing it by a four-tailed bandage.

Teeth consist of enamel, dentine, and cement; there are 10 milk teeth to each jaw, and 16 permanent; the latter are 4 incisors, 2 canines, 4 bicuspids, and 6 molars. They are cut in the order shown in the table:—

| | Incisors | | Canines | Molars | | | | |
|---|---|---|---|---|---|---|---|---|
| | Central | Lateral | | 1st | 2nd | | | |
| Primary set.. | 7 | 9 | 18 | 12 | 24 | | | —th month |
| Permanent set | 7 | 8 | 11 | 9 | 10 | 6 | 12 | 20th year |
| | | | | 1st | 2nd | 1st, | 2nd, | wisdom |
| | | | | Bicuspids | | Molars | | |

A tooth is composed of crown, neck, and root, the roots of the upper molars have three fangs, the lower two, but in wisdom teeth the fangs are merged in one. In rickets the teeth come late and go early, and often have defective enamel. In congenital syphilis the central incisor teeth are sometimes notched.

The palate is partly hard (bony) and partly soft (muscular), and is developed like the face in two symmetrical halves which coalesce with a median vertical septum. If this coalescence fail, hare-lip, and cleft of hard, or of the soft, or of the whole, palate may result, necessitating a plastic operation for its cure.

### To Nurses.

We invite contributions from any of our readers, and shall be glad to pay for "Notes on News from the Nursing World," or for articles describing nursing experiences, or dealing with any nursing question from an original point of view. The minimum payment for contributions is 5s., but we welcome interesting contributions of a column, or a page, in length. It may be added that notices of entertainments, presentations, and deaths are not paid for, but, of course, we are always glad to receive them. All rejected manuscripts are returned in due course, and all payments for manuscripts used are made as early as possible after the beginning of each quarter.

### Functions of the Week.

### DISTRIBUTION OF PRIZES TO THE PROBATIONERS AT THE LONDON HOSPITAL.

On Wednesday afternoon the probationers of the London Hospital received, from Sir Henry Roscoe, the prizes and certificates awarded on their examination. The occasion was a festive one, the successful students of the medical school also receiving prizes and certificates, and a large number of friends filled the Library of the Medical College and encouraged the recipients with cheers and applause. Sir Henry said it gave him even more pleasure to give prizes to the nurses than to the students, and Mr. Sydney Holland said that the record had been broken on this occasion, since every one of the 113 probationers had passed the examination.

The following were presented with prizes and certificates:—

1st Prize.—Probationer Kathleen Sybla Miller.
2nd Prize.— „ Ethel Annie Johnson.
3rd Prize.— „ Jessie Cairns.

Honorary Certificates.

*Probationers.*—Sarah Emily Parkes, Ethel Morris, Annie Katharine Randell Blandford, Minnie Fernley, Hannah Coope, Mary Alice Walmsley, Edith Marion Wenmoth, Edith Mary Harrison, Millicent Charlotte May Jackson, Nora Mortimer Gould, Gertrude Sarah Hay, Mary Wilhelmine Hein, Elsie Mussett, Sarah Ann Hephzibah Moss, Fanny Alice Harris Ireland, Grace Addenbrooke, Margaret Calverley Osmond, Alice Wainwright, Eileen Louisa Ellis, Annie Christabel Harwood, Gladys Mary Turner, Janet Elizabeth MacKenzie, Agnes Parsons, Charlotte Elizabeth Ann Glossop, Ida Day Barker, Clara Elizabeth Evans, Annie Frances Wade, Mary Beatrice Bateman, Elizabeth Norrish Lee, and Ada Dorothea Douglas.

The examination was in three parts, a paper was set, practical work in the wards was tested, and there was a *viva voce*. Mr. Holland said that wild horses would not drag from him the name of the probationer who had said, in answer to the question how to disinfect a room, that the room should be sealed up as soon as the case was diagnosed, and that the treatment should be continued until the patient either recovered or died; or of the one who, being asked how to remove the moisture from a man rescued from drowning, said she should hold him up by the heels and let the water run off. The "examination face" was one which he well knew, and he was equally familiar with the expression of relief that prevailed when the examiner had departed. The examiner was in the unusual position of being very popular on his departure, as well as on his arrival. An important alteration had been recently made by which each sister had only 30 patients, instead of 65, under her charge: this was in order that she might have more time to devote to nursing, while the burden of administration would be lessened. The example of the matron in devotion and unselfishness would thus, through the sisters, be more impressed upon the nurses and probationers.

After the prize-giving a musical programme was performed in the grounds by the Lawler Quintette and Miss Katherine Purcell.

### INSPECTION-DAY AT ST. PANCRAS INFIRMARY.

Once during the summer the ratepayers of St. Pancras have a special opportunity of paying a visit of inspection to the Infirmary, Dartmouth Park Hill. On Friday last some hundreds responded to the invitations sent out, and a very pleasant garden party was the result. The afternoon was very warm, and after going through the wards and kitchens, etc., the guests sat about under the shade of the trees surrounding the nurses' tennis-court listening to the band of the Y Division of Police, four policemen, of Scottish birth, attired as pipers, giving selections on the bagpipes at intervals. Refreshments were provided in one of the wards, which was cleared for the purpose. The babies' wards appeared to attract the largest number of visitors. Every place was gay with flowers, and flags and banners added to the festive appearance of the building and grounds. Musical entertainments took place in the wards during the evening.

### In the Relapsing Fever Wards, Arthur Road Hospital, Bombay.

BY ONE OF THE NURSES.

IT was towards the end of November that the relapsing fever epidemic began to assume alarming proportions. On the 15th of that month our wards were nearly empty, but by the beginning of December we were full to overflowing. A second large ward was opened, then a third, till, before the end of March, several of the sheds in what had been the segregation camp, had to be utilised for the same purpose.

### PATIENTS FROM THE JAIL.

The first patients who came to us were from the jail, where the disease had broken out in a virulent form. The distress caused by the terrible famine had brought numberless waifs and strays into Bombay to seek for work, whilst the closing of the mills in and around the city itself for several days in each month had accentuated the poverty of the enormous labouring population. The natural result of these combined causes was a very considerable increase in petty crime and in the number of vagrants and beggars that at all times swarm in the crowded streets and narrow thoroughfares of the native town, and the jail was overfull of starving, sickly folk. They began to arrive at the hospital in very large numbers. First a batch of eighteen, followed the next day by one of twenty-four. Then day after day for many days, fifteen, ten, or twelve at a time, and long before they were fit to be discharged the starving people from the town were being brought to us. Poor wanderers from Guzerat and Kathiwar, speaking a different language, wearing a different dress, and in habits and temperament very different from the Marathas to whom we were accustomed.

### FIVE EPIDEMICS AT ONCE.

By this time the plague was once again rife, and the worst epidemic of smallpox known in Bombay for many years was devastating the city, while hand in hand with it went measles and chicken-pox. These five epidemics running together filled our hospitals for five months, and were succeeded by the outbreak of cholera, which reached its height in August and September, when it claimed its 120 victims a day.

### RELAPSING FEVER.

A ward full of relapsing-fever patients in the acute stage is almost as terrible as a plague ward, except that the chances

Female. Aged 45. Coolie. Marathi Caste. Ratabar.

RELAPSING FEVER.

of recovery are very much greater and may be put down in a normal year at 85 per cent. The primary attack of fever usually lasts from three to five days. Then the temperature suddenly drops eight, ten, or even twelve degrees in a few hours, and the patient lies in a state of extreme collapse frequently succeeded by one of violent delirium. While this lasts he usually refuses all nourishment and medicine and needs the greatest care. The temperature generally remains sub-normal for seven, ten, or even twelve days, then suddenly runs up again to drop after three or four days in the same alarming way. Sometimes there is no second or third attack, but it occasionally happens that a patient gets as many as five attacks. The chart here reproduced is very typical. The patient had no further relapse, and was discharged cured.

### A VERY INFECTIOUS MALADY.

Though not nearly so fatal as plague, relapsing fever is far more infectious. Our ward attendants and sweepers, who seem to possess almost an immunity from the former disease, are constantly attacked with the latter. The famine-stricken and badly fed amongst the population are the first to contract it. They soon infect the healthy, and whole families are brought to us, all victims of this strange disorder. In cases considered difficult of diagnosis specimens of the blood have been frequently tested, when the discovery of the spirillum removed all doubts. Occasionally, the presence of both the spirillum and plague bacillus have been proved in a single specimen—the temperature charts of such patients showing the course of both diseases. The face of a person suffering from relapsing fever exhibits an appearance of considerable distress. The eyes are wide open, and the expression wild and haggard; the conjunctiva is yellow, sometimes quite orange-coloured; the tongue brown and glazed, with red edges; the breath foul. There is a peculiar odour about acute cases which baffles description but is easily recognisable. As the temperature falls the patient frequently suffers from diarrhœa and vomiting.

### NORMAL CASES.

The treatment of normal cases is simple enough. Whilst the fever lasts we rely on wet packs, hot or cold, ice, and a fever mixture to bring down the temperature; during the collapsed stage on stimulants, given either by mouth or by means of hyperdermic injections, hot bottles, and constant nourishment; whilst during the convalescent stage, between the attacks, we feed the patients well and give tonic mixtures.

### THE WARDS.

A brief description of the wards in which we worked may be of interest. They were built of bamboos and mats whitewashed within and without. Square holes furnished with mat shutters were cut at intervals for windows. The mat walls were raised about a foot from the floor and the intervening space could also be closed by shutters. The whole of the top under the roof was open, thus allowing of free ventilation. The floors left much to be desired, for being of mud they were very difficult to keep clean, even with a liberal use of disinfectants, and as in many cases they were rough and uneven they added considerably to our fatigue. These walls collapsed during the last monsoon so the relapsing cases are now accommodated in the fine pucca building.

### THE NURSES' WORK.

Each ward held forty beds and each sister had charge of two wards and of one or more of the camp huts in addition. As only one trained nurse was on duty at a time we had no lack of hard work. We are assisted by ward-boys and ayahs. After a little teaching many of the men make excellent nurses. They are intelligent, fond of learning, and ready to do anything to please the "sister sahibs." At the same time they require perpetual supervision, for unless one is looking they will always take the, to them, easiest way of doing things. When first they came to us they were only raw coolies, fresh from the mills, and their improvement is really wonderful. But even now, though he knows perfectly well how to wash a patient, the ward-boy will still bring a feeding-cup for that purpose! Pouring a little water from the spout into the hollow of his hand, he will smear it over the patient's face, apparently imagining that he has done all that can possibly be required of him. When found fault with for such laziness, no matter how sharply, he will smile in the most deprecating and engaging manner, and go off with perfect good humour to fetch a "guindy" (hand-basin) of water and a sponge, with which he proceeds to do his work properly at last.

*(To be continued.)*

### Nursing in Syracuse, N.Y.

By An American Graduate.

I wonder whether the editor of the Nursing Mirror can "guess"—(no, that is too American)—can "imagine" how warmly the paper is welcomed, and how eagerly all written therein is perused. The, to me, familiar names, the items of professional interest, the notes and letters from our far-scattered sisters are all read enthusiastically, and I think perhaps some might care to know a little of what a graduate nurse's life in Syracuse is like.

"Just in, and resting." To most of those who read these words their inner signification will be revealed as they cannot be to others whose ministrations to the sick are confined to the mere utterance of sympathy. The history of nursing is, I presume, largely the same in all lands. We go in and out among all sorts and conditions of humanity, often the vital part in our environment, seldom indeed of it, taking up the burden of life where the weak ones perforce had to lay it down. Then, when the mission is accomplished, passing out into the welcome shadow and rest of a room where God's angels only understand what the personal cost of our labour has been.

### The Club and Surroundings.

Our club here is a large family residence, where twenty-three members are presided over by a motherly woman in charge of all the essential details of comfort. Individual taste is exercised in the matter of room decoration and furnishing, and is a happy idea, for an element of "home" feeling is thus added. The house is beautifully situated on one of the principal streets. To our right lie the green lawns of the orphanage, where 200 little pink-and-white mysteries with a small percentage of colour are daily growing more and more complex problems for a paternal government to solve. Poor wee waifs and strays who, born in hospitals or, as far as they understand, not "born" at all, simply "growed" like Topsy, are received at the age of two years and maintained until able to do something for their personal support. Next to us rises the spire of a large Methodist church. The University here is mainly composed of Methodists, and their places of worship are the largest and best attended. Opposite is the square Norman tower of the Episcopal church, usually designated the English church. I love to slip in there, and take a part in the dear old service. It is a link which draws me across the seas to those other services, held long ago, when mother and brother's presence meant life and love and all the world to me. Beyond and around us the blue veiled Onondaga hills mount skywards. The Indian word Onondaga means two hills and a valley.

### The Busy Season.

There are nearly three hundred graduate nurses in this city, the majority of whom are noble, earnest women, loyal to their school, but not bigoted. Between them and the doctors is a cordial friendship which redounds to the credit of both sides. From September to May or June there is nearly constant work for a good nurse. In June the city commences to empty fast. Families retire to the north woods, to the seaside, or among the lovely Thousand Isles, and nurses go to find in home, or camp, or beach, that change which is essential to health. About August the influx begins. There is a pulsation of new life in the city; students and pleasure-seekers come strolling back to duty. House-shades that had been tightly drawn are raised; glistening stars and stripes hang from windows, enclose piazzas, form tents, and decorate flag-staffs. You hear merry welcomes shouted across streets, and the little buzzings of enthusiastic interchange of summer experiences only die softly away when the first whisper comes up that the boy indulged in too many green plums and has "an awful pain under his pinafore."

### An Epidemic of Typhoid.

Last autumn, in the State Asylum, an epidemic of typhoid occurred, which the hospital authorities were unable to cope with alone. I was with them for fourteen weeks, and out of thirty-six cases I did not lose one, though several of my patients had pneumonic complications. The doctor had the fever more severely than any case I have ever met with. Five physicians gave up hope, but we worked on, and to-day he is well and leading an active life.

### A Fight for Life.

I had a very interesting case in December. An old lady with a scorbutic trouble of twenty years' standing, and a most pronounced tendency to diabetes, had a gangrenous toe, heel and ankle. This was another case pronounced as hopeless She desired to know her condition, and her physicians told her. Late that night she took my hand in hers and asked, "Do you think I must die?" There was a world of agony in the question; a close clinging to a pain-wrecked life that was mysterious to me. But she held to the words of hope with such a tight clasp that the very cleverest surgeon in the city had something tangible to build on, and though it meant weeks of hard fighting, we won, and that old lady is sitting as I write by an open window on her couch enjoying the sweet fresh air as it sweeps over the hills to her.

### A Dear Little Patient.

It is for our little ones my heart grows most tender—the dear little mites whose cross of suffering is borne with such loving heroism and such faith in us that the more I see the more I marvel at it. A wee girl had been bound body and feet to a curass for three years of her small life, and when at last she was told that the bandages were coming off there was something almost holy in the joy of the whole nursery over her emancipation. The first time the pet was lifted she turned in her nurse's arms and stretching out her hands to the curass patted it with her soft palms as if it were a living friend and lisped, "Don't be thorry and lonely for me, bruther. You've been nithe to me, and I'll love you alwayth. I'll be back thoon." Up to the day she left us she kissed that curass every night. Who among us would cultivate feelings of gratitude for that which had been a daily penance for three years of our life?

### Queen Victoria's Jubilee Institute for Nurses.

HER MAJESTY QUEEN ALEXANDRA has been graciously pleased to approve the appointment of the following as "Queen's Nurses," to date July 1, 1901:—

ENGLAND.

| Nurse | District Training at | Working at |
|---|---|---|
| Emma M. Greenwood | Central Home, London | Cumberland (superintendent) |
| Edith K. Hewlett | Hammersmith | London |
| Maud Gent | Southampton | Silvertown |
| Nellie Blew | Battersea | London |
| Annie Dyer | Ditto | Ditto |
| Lucy E. Way | Central Home, London | Ditto |
| Annie J. Dashwood | Paddington | Hounslow |
| Clementina Methuen | Woolwich | Woolwich |
| Emily Freeman | Chelsea | Portsmouth |
| Caroline S. Goode | Ditto | Accrington |
| Margaret Hardman | Westminster | Chatham |
| Lilian Trendell | Battersea | Ditto |
| Florence Goode | Brighton | Brighton |
| Georgina M. Laming | Ditto | Ditto |
| Elizabeth M. King | Ditto | Ditto |
| Catherine Benn | Liverpool | Liverpool |
| Olive Pound | Ditto | Ditto |
| Edith A. Morley | Ditto | Ditto |
| Rebecca Wright | Ditto | Ditto |
| Minnie Willis | Ditto | Ditto |
| Mary Ellen Hopwood | Ditto | Ditto |
| Emma A. S. Pilgrim | Birmingham | Birmingham |
| Emma M. C. Paine | Ditto | Ditto |
| Clara Saunders | Ditto | Ditto |
| Esther G. Evans | Ditto | Ditto |
| Emily Jackson | Salford | Salford |
| Mary Carsley | Ditto | Ditto |
| Margaret Turner | Manchester | Manchester |
| Ellen Eckersley | Hull | Hull |
| Annie Horrocks | Manchester | Hull |
| Ellen Harbridge | Gloucester | Gloucester |
| Hermione C. H. Blackwood | Central Home, London | Ditto |
| Charlotte Webster | Liverpool | Gloucester |
| Mary Lumsden | Windsor | Windsor |
| Jessie E. Turnbull | Tunbridge Wells | Tunbridge Wells |
| Mabel L. Everard | Haggerston | Burnham |
| Margaret E. Akers | Ditto | Wormbridge |
| Annie Geoghegan | Ditto | Stamford |
| Gertrude Lawton | Ditto | Darwen |
| Marion A. G. Smythe | Central Home, London | Matlock |
| Mary Tewson | Ditto | Amarsham |
| Hannah P. Longford | Ditto | Millom |
| Kate Parfitt | Ditto | Bilston |
| Helen W. Clydesdale | Ditto | Shildon |
| Emily A. Cross | Ditto | Loughborough |
| Elizabeth Singer | Bermondsey | Newbiggin-by-the-Sea |
| Lilian Menzies Jackson | Walworth | Braughing |
| Edith M. Steward | Portsmouth | Strood |
| Florence S. Packard | Ditto | Bridgwater |
| Minnie M. Davey | Ditto | Upton-on-Severn |
| Alice Jane Sanders | Camberwell | Totnes |
| Harriet E. Grattan | Brighton | Edensor |
| Kate G. McKenna | Brighton | Quedgeley |
| Annie Everall | Walworth | Dartmouth |
| Clara Brown | Gloucester | Haslingden |
| Elizabeth Nield | Salford | Bilston |
| Alice A. Jones | Liverpool | St. Helens |
| Mary A. Yeats | Liverpool | Droylsden |
| Ellen P. D. Saingear | Cardiff | Gainford |
| Rebecca Haddon | Hunton | Hunton |
| Margaret Roberts | Edinburgh | Harpenden |
| Helen Higgs | Coventry | Coventry |
| Florence Warburton | Castor & Sutton | Castor & Sutton |

WALES.

| Nurse | District Training at | Working at |
|---|---|---|
| Elizabeth Butterworth | Haggerston | Cardiff |
| Harriet Austen | Cardiff | Treorchy |
| Eva I. Trill | Bermondsey | Llandilo |
| Edith M. Adams | Cardiff | Rhayader |

WALES—*Continued.*

| Nurse | District Training at | Working at |
|---|---|---|
| Catherine Jones | Central Home, London | Harlech |
| Kate Hughes | Liverpool | Broughton |
| Agnes McClure | Portsmouth | Conway |
| Sarah Hughes | Bermondsey | Llanfairfechan |
| Mary Freeman | Brighton | Ruthin |

SCOTLAND.

| Nurse | District Training at | Working at |
|---|---|---|
| Mary C. McGillevray | Edinburgh | Edinburgh |
| Jessie M. Yorks | Ditto | Ditto |
| Elizabeth M. A. McCulloch | Ditto | Ditto |
| Marianne McLean | Ditto | Ditto |
| Eleanor G. Turnell | Ditto | Ditto |
| Elizabeth Robertson | Ditto | Ditto |
| Margaret C. Bayley | Ditto | Ditto |
| Emily Fraser | Ditto | Ditto |
| Thomasina Purves | Ditto | Ditto |
| Margaret M. A. Williams n | Ditto | Ditto |
| Annie M. Newman | Glasgow | Glasgow |
| Helen Gordon | Ditto | Ditto |
| Adelaide Whieldon | Edinburgh | Kirkcaldy |
| Eliza Barrett | Ditto | Ditto |
| Agnes Pike | Edinburgh | Tobermory, Mull |
| Margaret A. C. Smith | Ditto | Polmont |
| Christina Honison | Ditto | Bothwell |
| Alice A. O. Touch | Ditto | Glencorse |

IRELAND.

| Nurse | District Training at | Working at |
|---|---|---|
| Kathleen Blake | Dublin | Dublin |
| Annie Smithson | Ditto | Ditto |
| Margaret L. Fryer | Ditto | Ditto |
| Anna Forster | Ditto | Ditto |
| Eunice Patrick | Ditto | Ditto |
| Annette Fitzgerald | Ditto | Ditto |
| Jane Dodwell | Londonderry | Londonderry |
| Frances Elliott | Ditto | Ditto |
| Margaret F. Noblett | Dublin | Crumlin |
| Emily Gillespie | Ditto | Mallow |

### Appointments.

BELPER ISOLATION HOSPITAL.—Miss Theresa M. Gilburns has been appointed superintendent nurse. She was trained at East Dulwich Infirmary, and has since been night superintendent and sister-in-charge of male wards at Tunbridge Wells General Hospital.

BRITISH LYING-IN HOSPITAL, ENDELL STREET, LONDON, W.C.—Miss Gertrude Knott has been appointed matron. She was trained at the Children's Hospital, Pendlebury, and Guy's, where she subsequently held the post of night sister for a year. She has been assistant matron at the British Lying-in Hospital since April, 1899. She holds the certificate for midwifery of the British Lying-in Hospital and the L.O.S. certificate.

COUNTY ASYLUM, RAINHILL.—Miss Hilda Hewitt Lawrence has been appointed charge nurse of the Phthisis and Isolation Hospital attached to the Asylum. She was trained at the Fir Vale Infirmary, Sheffield Union, and has since been nurse at Huddersfield Union Infirmary and Chester Union Infirmary.

GENERAL INFIRMARY, MACCLESFIELD.—Miss Berry has been appointed sister of the children's ward. She was trained at the Royal Infirmary, Preston.

PADDINGTON GREEN CHILDREN'S HOSPITAL.—Miss S. T. Biddulph Pinchard has been appointed matron. She was trained at the East London Children's and Charing Cross Hospitals. She has since been matron of Princess Mary Convalescent Home, Bognor, a branch of the East London Children's Hospital.

PONTEFRACT JOINT HOSPITAL.—Miss Helen Hodgson has been appointed head nurse. She was trained at Suffolk General Hospital, Bury St. Edmunds.

SIERRA LEONE NURSING HOME.—Miss I. M. Johnston has been appointed temporary nurse. She was trained at Guy's Hospital, and at the Maternity Charity, Plaistow. She holds the L.O.S. certificate.

### Echoes from the Outside World.

### AN OPEN LETTER TO A HOSPITAL NURSE.

THE sum subscribed towards the National Memorial to Queen Victoria is exceedingly disappointing. It was generally expected that at the end of June something approaching a quarter of a million of money would have been contributed by a devoted people. But the amount now in the hands of the committee is less than half the total anticipated. Of course, there are differences of opinion among the committee as to the appropriateness of the numerous schemes submitted to them. One is by Mr. Lennox Browne, the throat specialist, who suggests the adaptation of Buckingham Palace to the purposes of a memorial by the addition of a storey at each end, the erection of a lofty central roof, and the facing of the building in red brick and stone. According to Mr. Browne's plan, this would make a background for a great avenue leading from the eastward of Piccadilly, opposite Devonshire House, to the Palace, in the line of the present footpath; and he further suggests an opening into Trafalgar Square. The forecourt of the Palace, he proposes, should be extended into a great semicircle with arches emblematical of Great Britain's Colonies. The scheme is interesting and ingenious, but it is very elaborate, and would be very costly. It seems to me that until the committee know how much money they are going to receive they cannot come to any decision. But that only a little over £100,000 should have been promised in six months either shows that things have been badly managed, or that the British public are more effusive than grateful.

---

IT is very doubtful whether more than an extremely limited number of women desire to possess the Parliamentary franchise. When the number becomes great, my belief is that the demand will prove irresistible. Women have a knack of getting their way when they are really in earnest. But it is curious that the measure brought forward in the House of Lords last week to allow women to sit on the London borough councils, as they were permitted to sit on the old vestries, should have been as hotly opposed as if it necessarily involved the concession of the suffrage. The Bishop of Rochester put the case with unanswerable force when he said that "in the poorer districts the number of people who have the leisure, the education, and the breadth of mind to deal in the best spirit with municipal administration must necessarily be small, and the exclusion of women means the exclusion of a very valuable contingent." But Lord James of Hereford, who made a speech that might have been delivered with fitness when he was playing with ninepins, drew a picture of the awful consequences of the insertion of the thin end of the wedge, which so frightened the poor peers that even the Archbishop of York's appeal to them to vote "undeterred by terrible difficulties apprehended to arise in the remote future" had no appreciable effect, and women, for a time at any rate, are to be shut out from work which is particularly suitable for them.

---

NATAL may not be a very large colony, but assuredly it is a go-ahead one. At Durban and Maritzburg the education of mind and brain has always taken a very important place, and now it appears that the public schools of the seaside town are determined to be in no wise behind the home colleges in the physical training they are able to offer their pupils. The principal of one of the largest schools in Durban has lately been in England, with the view of engaging a games and gymnastic mistress. The young lady who has undertaken the position is Miss Edith Brown, who was trained at Southport Physical Training College. Although she is only twenty-one years of age she has already secured in the acquisition of the accomplishments to which she has devoted her energy almost all the honours possible. She is stated to be qualified in physiology, hygiene, sick nursing, and ambulance work, which, I conclude means First Aid, etc. She has also been instructed in medical gymnastics, has obtained a gold medal for practical gymnastics, a gold medal for climbing a rope 60 feet high, and a gold medal for swimming one mile. She can fence, vault nearly her own height, and play cricket, hockey, and other outdoor games. She is to be paid £300 a year, which is a liberal salary as emoluments go, but it must be remembered that athletics in the winter in Durban, when the heat is intense, are much harder work than they are in England, and therefore, naturally, the instructress is deserving of higher remuneration for the extra fatigue. Miss Brown will not find much difficulty, I fancy, in getting up enthusiasm amongst her pupils. The Natalians whom I know put many English girls to shame in the amount of hard work they get through. They seem to do a great deal in their homes—owing to the difficulty of getting dependable black servants—to sew and cook and dust all the morning, entertain friends to lunch or dinner almost daily, play tennis all the afternoon, and dance all the evening. Girls who can do this all the year round, ought to prove good athletes.

---

THE Exhibition of the Pastel Society at the Royal Institute of Painters is just the kind to please towards the end of the season when one is tired of more substantial fare. There is something dainty and restful about Pastel painting; it seems to require less brain power to enjoy it than either water-colour or oil painting, and nurses will be glad of half an hour's refreshment in the galleries in Piccadilly. There are a great many contributions by foreigners, doubtless, because, as a rule, they shine more in this particular form of art than the average Englishman or Englishwoman. The works by M. René Billotte are especially worthy of notice, including "Feux d'Octobre," a simple realistic transcription of autumn fires consuming weeds and useless rubbish; and "Lever de Lune à Meulan," representing a solitary marsh, cheerless and sad, in whose dismal waters the rising moon is just beginning to reflect itself, giving them a strange, almost unearthly, beauty. M. Nozal has an unusual picture, "The Soir-Canal du Loing," which is brightened by a violet sunset. Mr. George Clausen has a beautiful "Thresher," Mr. Henry Tuke a "Woodland Study," a sunlit wood, half concealing the figure of a nude youth, and Mr. Joseph Pennell sends two pictures which form a clever contrast to each other, "A June Afternoon," green and yellow, dull blue, grey and purple, hues which are sufficiently subdued in themselves, and yet so subtly are they blended that they convey in a marvellous manner the very atmosphere the artist intended, and the other "Winter on the Thames," all black and white, cold and drear. Mr. Blake Wirgman succeeds in showing how excellent a medium pastels can be for portrait painting in a first-rate study of a blonde lady, 'Mrs. Hemming."

---

A FEW years back all the sales at the big shops began on July 1st or the first Monday in the month, and those who were anxious to be early in the field could only hope to "do" two, or at most three, shops the first day. Now the largest establishments arrange for their dates not to clash with each other, so that if one date is inconvenient, it is easy for purchasers to wait and go somewhere else on another day. Two at least of the leading houses begin their sales next week. This year more can be effected than usual by a hoarded little sum of money judiciously spent, because black and white and grey have been so universally worn that much coloured material of value has been thrown on the shopkeepers' hands, and this has been, and will be, sold at a low price. Nurses with clever fingers will do well to buy some odd lengths of ribbon and lace. It is so usual now to wear different detachable neck-bands with blouses that a great many are required, and a great many, if made at home, can be procured for almost a song. Short lengths of wide handsome ribbon can be advantageously utilised for waist-belts, either swathed or draped and sewn on to a bone, four or five inches deep at the back. The front can be finished by a deep buckle, tying ribbons, or handsome buttons and corded loops. I hear that owing to the fact of brown having had such a back place in the affections of the society lady of late, it will be received into favour again this autumn. I give the rumour for what it is worth, but it may act as a guide to those anxious to buy themselves something serviceable and yet up to date in the way of a gown for the early winter.

### Everybody's Opinion.

[Correspondence on all subjects is invited, but we cannot in any way be responsible for the opinions expressed by our correspondents. No communication can be entertained if the name and address of the correspondent is not given, as a guarantee of good faith but not necessarily for publication, or unless one side of the paper only is written on.]

### AN APPEAL FOR HELP.

"SOPHIE" writes: Will any nurse kindly help me in the following matter? I have a patient who is suffering from chronic rheumatic gout, and the lowest vertebra of the spine is unusually prominent. This has always caused her some inconvenience when in health, but now that the surrounding tissue has become wasted through illness, and being unable to take any exercise, the pain is often very great, and sometimes it seems impossible to procure any rest for her, either lying down at night, or when sitting up in a large easy-chair by day. I always attend most carefully to the back both night and morning, rubbing it with spirit and applying boric powder, and there is no redness, and the skin appears perfectly healthy. Also I have consulted the doctor in attendance, but the lotion which he has prescribed only gives temporary relief, and the pain returns after a very short period of sitting. Of course I have tried an air ring, but this causes such aching and pain to the legs that it has had to be given up. I think all nurses will sympathise with me in being unable to relieve pain, and if anyone will give me some advice I shall be deeply grateful.

### NURSING BY RELIGIOUS ORDERS.

"FAIR PLAY" writes: As to the remarks made in the NURSING MIRROR on the subject of the nursing in "religious" hospitals, both Catholic and Nonconformist, I wish to state that I hold a certificate and testimonials from a first-class provincial training school in England, a very secular one, I have also had good experience as a nurse on the "other side." But my own experience is that the time out of the ward that a sister (or medical mission woman) spends in devotion does not average more than the ordinary time the ward-sister spends in lawful recreation, in tea-parties, cycling excursions, half-days off, etc. One cannot fetch a ward-sister back from her "evening out" if she has started, but sisters can always be fetched out of chapel. Also, the latter are often on duty earlier and later than seculars—and in the English Church (and I believe among dissenters) are nearly always well-qualified women of the best stamp. Surely it is a matter for private decision how they spend their "off-duty." I write with many apologies, only because those who have the best right to speak will probably be silent on their own behalf.

### NURSING SMALLPOX IN A COTTAGE.

"E. G. B." (a district nurse) writes: You may, after the recent article on nursing smallpox at Glasgow, think my experience of nursing smallpox in a country cottage sufficiently interesting to publish. It was a low thatched cottage, about fifteen miles from the nearest town; picturesque outside, but, alas! sadly wanting in ventilation and sanitation within. I found it very difficult to get anything for my patient. The doctor who attended kindly brought me many little things from town, and had it not been for him I do not know how my patient or I would have fared, as we were almost boycotted by neighbours. The people were so afraid of infection that they would hardly pass the door, unless obliged to do so. My patient's husband assisted me in nursing his wife, and he used to throw money out into the road for passers-by to buy him some tobacco. They would not come near him, but placed the tobacco on the gate-post, at the entrance of the garden. Another thing he had to do was to fetch water from a neighbouring pump and often to lift the water over the wall to me on the other side, because the people would keep their garden doors locked for fear we should go too near. One day we had run out of coal, and I sent the husband to the nearest village (three miles distant) for some. He came back without, saying the people fled from him. So there was nothing to do but pick up sticks and use them as long as we could. Fortunately, our good doctor came that day, and when he heard of our dilemma kindly undertook to order the coals for us and had them sent at once. My patient was a woman about thirty years of age, and she had three children, all of whom were taken to relatives. But it was not till my arrival that the youngest was sent away. The grandmother was very obstinate about taking it, and only after the doctor assured her that I should be sent back to hospital if she did not comply with his request did she and the child depart. Oh! the filthiness of that place! I know I made a memorandum of these lines:—

> "The cottage was a thatched one,
> The outside old and mean,
> Yet everything within that cot
> Was neither neat nor clean."

Of course I had all the cooking and cleaning to do as well as the nursing. But worst of all was that poor patient. Never before had I seen such an object. She had just had an abortion, and was in a very weak state. Her face was so swollen one could not recognise the features. In fact, her whole body was covered with pustules. Eyes and mouth were very bad. The first thing we did was to carry her upstairs; she was then on a bed in the kitchen. We made her as comfortable as possible—her husband and I were the only ones to do anything for her. Then I began to turn out and clean the kitchen and sprinkled carbolic about. Everything was washed in a solution of carbolic, even to tables and chairs, and by the time I had finished the place smelt sweeter. The time of year was corn harvest, and the season was very hot. I made the most of the little windows, and left them open day and night, with a draught through: to this I mainly attribute my escape from infection. Of course I was vaccinated at once, and so also was my patient's husband. My patient seemed to improve till the eighth day of my being there, when the pustules were drying up. Then suddenly she was taken worse. The fever ran high, till it attained 108°. I sent off for the doctor at 3 A.M.; but, alas! the man returned about 6 A.M., without him. I waited, trying all I could to reduce the temperature with cold cloths wrung out of water with a solution of carbolic, and also touching the sores with a feather dipped in carbolic oil. The hours dragged on till 12 noon, when the doctor arrived, but too late to preserve life. He told me the disease was of such a malignant and confluent form that it would have been impossible to save her. It had gone internally, and we believe the fœtus had not come away; so this also was poisoned, adding to the mother's suffering, and resulting in death. I am thankful to say I was perfectly well all the time I was there, and no one took the disease, except the patient's two brothers, who attended the funeral, and they only had it in a very mild form.

### Presentations.

ROYAL INFIRMARY, PRESTON.—Miss Berry, before leaving for her new appointment as sister of the children's ward at Macclesfield Infirmary, was presented with an afternoon tea service by the sisters and nurses of the Preston Infirmary.

CITY OF GLASGOW FEVER HOSPITAL.—Last week Miss Cunningham, the acting matron of Kennedy Street Hospital, was the recipient of a handsome silver afternoon tea service from the medical and nursing staff on the occasion of the resignation of her appointment in view of her approaching marriage. The presentation was made by Miss Adams, matron of Ruchill and Kennedy Street Fever Hospitals, who expressed the esteem in which Miss Cunningham is held by the nurses and all who have come into contact with her in the discharge of her duties, and who took the opportunity to wish her every success in her new sphere.

### For Reading to the Sick.

TAKE THOU MY HAND.

A TENDER child of summers three,
Seeking her little bed at night,
Paused on the dark stair timidly.
"O Mother! take my hand," said she,
"And then the dark will all be light."

We older children grope our way
From dark behind to dark before;
And only when our hands we lay,
Dear Lord, in Thine, the night is day,
And there is darkness nevermore.

*Whittier.*

I cannot say,
Beneath the pressure of life's care to-day,
I joy in these;
But I can say,
That I had rather walk this rugged way,
If Him it please.

I cannot feel
That all is well, when dark'ning clouds conceal
The shining sun;
But then I know
God lives and loves; and say, since it is so,
Thy will be done.

I do not see
Why God should e'en permit some things to be,
When he is love;
But I can see,
Though often dimly, through the mystery,
His hand above.—*S. G. Browning.*

A heart rejoicing in God delights in all His will, and is surely provided with the most firm joy in all estates; for if nothing can come to pass beside or against His will, then cannot that soul be vexed which delights in Him and hath no will but His, but follows Him in all times, in all estates; not only when He shines bright on them, but when they are clouded. That flower which follows the sun doth so even in dark and cloudy days: when it doth not shine forth, yet it follows the hidden course and motion of it. So the soul that moves after God keeps that course when He hides His face; is content, yea, even glad at His will in all estates or conditions or events.—*R. Leighton.*

It is a blessed secret, this of living by the day. Anyone can carry his burden, however heavy, till nightfall. Anyone can do his work, however hard, for one day. Anyone can live sweetly, quietly, patiently, lovingly, and purely till the sun goes down. And this is all that life really ever means to us—just one little day. "Do to-day's duty, fight to-day's temptation, and do not weaken and distract yourself by looking forward to things you cannot see, and could not understand if you saw them." God gives us nights to shut down the curtain of darkness on our little days.

*J. R. Miller, D.D. Secrets of a Beautiful Life.*

Lead me, O Father, holding by Thy hand;
I ask not whither, for it must be on.

*George MacDonald. Within and Without.*

### Notes and Queries.

**The Editor is always willing to answer in this column, without any fee, all reasonable questions, as soon as possible.**

**But the following rules must be carefully observed:—**

1. **Every communication must be accompanied by the name and address of the writer.**
2. **The question must always bear upon nursing, directly or indirectly.**

**If an answer is required by letter a fee of half-a-crown must be enclosed with the note containing the inquiry.**

*The Finsen Light Treatment.*

(137) Mr. Sydney Holland writes to us saying that the only other Finsen lamp in England, besides the apparatus in use at the London Hospital, is one in the possession of Dr. Blackie. Probably he means Dr. Blacker, Superintendent of the X-ray Department at St. Thomas's Hospital, who, we understand, was the first to introduce the treatment into London.

*Dispensing.*

(138) I am hoping to obtain an appointment as nurse to a mission in South Africa. One of the requirements is a knowledge of dispensing; and I am told that three months would be sufficient time in which to prepare for it. Can you tell me of a dispensary in the north of London where I could learn?—*L. H. L.*

Perhaps your local chemist would instruct you. The Secretary of the School of the Pharmaceutical Society, 17 Bloomsbury Square, W.C., might give you the required information.

*Stone Deaf.*

(139) I should be glad if you can kindly tell me of any home or institute where an old man, nearly stone deaf, could be received. His health is good, but he is incapable of work. His friend would pay 7s. to 10s. weekly for him.—*Sister Maude, Cardiff.*

The Little Sisters of the Poor, St. Peter's House, Meadow Road, South Lambeth, S.W., seems the most likely. Perhaps one of our readers could recommend a home.

*Male Nurse.*

(140) Can you tell me if there is any hospital in the British Isles for training male nurses?—*J. R. S.*

There is no hospital where male nurses only are trained. The National Hospital for the Paralysed and Epileptic, Queen's Square, Bloomsbury, W.C. offers male nurses a two years' course of instruction.

*Certificate.*

(141) Will you be kind enough to tell me if an association has a right to keep a certificate of monthly training? The nurse's fees were paid by the association upon her signing an agreement to work for it for three years. She could not continue her duties on account of ill-health.—*Brownie.*

Certainly. The association paid for the training, and as the nurse was unfortunately not able to keep her part of the contract she cannot expect her certificate.

*Drink Cure.*

(142) Would you kindly let me know what I could give a poor woman to satisfy the craving for alcohol? She is anxious to give up the habit.—*Anxious.*

You cannot cure the craving for alcohol by giving medicines; but possibly the Secretary of the British Women's Temperance Association, 47 Victoria Street, London, S.W., would be able to advise you as to the best method of dealing with the case.

*Re Maternity Nursing.*

(143) Would you inform me if nurses, not holding a midwives' certificate, are sent out to attend district maternity cases without the supervision of a doctor, by such hospitals as St. George's, the Middlesex, St. Bartholomew's, &c.?—*Nurse A.* Certainly not.

Is there any hospital or institution where free training is given in monthly nursing?—*A. E. M.* No.

The lady I was engaged to attend was prematurely confined, and died. May I claim my fees from her husband?—*A. H.* Yes.

Will you tell me if there is any law against a nurse with several years' experience in general nursing, and four years' practical work in a lying-in-ward (but who has not the L.O.S. certificate) taking private maternity cases amongst the poor?—*Nurse M. A. B.* No.

Please tell me at which institutions I can get maternity training for a fee of £7 7s.—*Constant Reader.* The British Lying-in Hospital, Endell Street, St. Giles, W.C., fee £7 3s., and the City of London Lying-in Hospital, City Road, E.C., fee £7 7s.

**Standard Books of Reference.**

"The Nursing Profession: How and Where to Train." 2s. net; post free 2s. 4d.

"Burdett's Official Nursing Directory." 3s. net.; post free, 3s. 4d.

"Burdett's Hospitals and Charities." 5s.

"The Nurses' Dictionary of Medical Terms." 2s.

"Burdett's Series of Nursing Text-Books." 1s. each.

"A Handbook for Nurses." (Illustrated.) 5s.

"Nursing: Its Theory and Practice." New Edition. 3s. 6d.

"Helps in Sickness and to Health." Fifteenth Thousand. 5s.

"The Physiological Feeding of Infants." 1s.

"The Physiological Nursery Chart." 1s.; post free, 1s. 3d.

"Hospital Expenditure: The Commissariat." 2s. 6d.

All these are published by THE SCIENTIFIC PRESS, Ltd., and may be obtained through any bookseller or direct from the publishers, 28 and 29 Southampton Street, London, W.C.

### Travel Notes.

By Our Travelling Correspondent.

### LXXV.—IN THE MOSELLE COUNTRY.

For those whose time is limited and purses slender, no continental locality within reasonable distance is more suitable than the valley of the Moselle. It has many advantages; not only is the journey short and therefore cheap, but living is simple. Not being as yet so over-run by tourists as the Rhine, the innkeepers of the Moselle country are more like hosts, and do much in a simple, kindly way to make you feel at home, and prices are very moderate.

### The Cost of the Journey.

If you can spare the time the cheapest and by far the pleasantest way is *via* Rotterdam by the Netherlands steamboats. We have a line of steamers from Blackwall and the Tower which run in connection with them; by this route, the cost second class return to Coblentz is £1 7s. The journey from Rotterdam will occupy two days in this leisurely and delightful way of travelling, therefore, if your holiday is short you must go by Harwich, the Hook, and then by rail, for which your ticket will come to £2 12s. 11d. I take Coblentz as your starting place, because any distance beyond that will be but little extra expense, and one cannot say which point you will choose. Coblentz stands most impressively at the junction of the Moselle and the Rhine, under the frowning fortress of Ehrenbreitstein, called the Gibraltar of the Rhine. Standing on its bastions it is very singular to look down on the meeting of the waters, one green and one brown, which distinctive colours remain unchanged for some distance.

### How to Explore the Neighbourhood.

Pension terms in this charming country may be had from 3½ marks, and the best of everything for 4 marks, so that a holiday lasting a month will only cost £6 as far as board and lodging are concerned. For this reason if economy is an object it will be wise to avoid Coblentz and Trêves, and stick to the small and charming villages that lie between. There are steamboats plying up and down the river which make excursions easy and cheap, but in a very dry season they frequently cannot get up, and that pleasant means of travelling is suspended. There is, however, always the railway, and by its help the rustic valley can be explored even by a very humble pedestrian. Among the numerous charming spots at which to stop it is difficult to choose, but I think Moselkern or Carden, Alf, and Berncastel make good resting places, and if you can spend a couple of days in Trêves, so much the better.

### Moselkern or Carden.

Of the two I fancy Moselkern is rather the prettier, but Carden is a steamboat station, which is a great advantage. The narrow valley of the Eltz opens up immediately behind Moselkern, and the great attraction is the truly magnificent castle called Schloss Eltz. Here you will see a mediæval castle in perfect repair and fitted up throughout in accordance with the different periods of the architecture; anyone may enter the courtyard and study the exterior of the building, but for the interior it is necessary to apply for a written permission a week beforehand, which your landlord will get for you. Should the family be in residence and the order perhaps refused, do not be too much cast down; it is the exterior that is so wonderfully fascinating, with its multitude of high-peaked roofs, set at every possible angle, its hundreds of chimneys and wonderful hooded windows that cling precariously to the overhanging walls and steep roofs. The most charming view of all is as you approach up the sharply-ascending road which must have been a most unpleasant experience if hostile archers were stationed on the walls. The walk to the Schloss is absolutely lovely, and not more than half an hour from Moselkern; from Carden it is further, but equally entrancing. Walk across from Carden to Eltz, and instead of returning the same way, descend into Moselkern so as to see both paths; if you are tired take the train home. What between steamboats, trains, and ferries, getting about is very easy.

Carden itself possesses an interesting church, with a fragment of fine cloisters and a good chapter house. St. Castor made Carden his dwelling-place and here he died, but his bones were removed to the Castorkirche in Coblentz. Not far from the station is a very interesting building of which I should like to find a thoroughly good account; they call it the Tithe House, and it must certainly be as old as the beginning of the twelfth century. There is, too, the Burghaus, built in the sixteenth century. Altogether little Carden has many attractions for the artist and antiquarian and if you have no tastes of this kind but love beautiful scenery, long walks, or cycling, I know no place that will better meet your requirements. Excursions are numerous among other places do not omit to visit Münster-Maifeld. Take the train to Hatzenport and there the diligence, or go on your own legs according to taste. It is a strange old town, once of very considerable importance but now quiet and sleepy. It has a grand church, very ancient and somewhat resembling a fortified dwelling in the front. The different parts are of varying dates, and it is well worth a visit. There is a very pretty walk from Münster-Maifeld home by Schloss Eltz, and I am almost sure you will wish to see the latter place more than once.

### Cochem and Alken.

Cochem is justly considered one of the prettiest places on the Moselle; it will make a nice morning excursion, or for the whole day if you explore the beautiful Ender-Thal. The views all round are superb, and there are two castles full of interest, that of Cochem itself, which was formerly the country house of the Archbishops of Trêves, and the old home of the Metternichs in the Ender-Thal. Both these buildings were destroyed in the weary wars with the French in 1689. The Cochem castle has been beautifully restored according to the old plans. I should not advise your spending money to see the inside. In the opposite direction lies Alken, a most picturesque spot, with its old towers rising above the village, a paradise for the artist, and with such walks all round. About a mile and a half nearer Carden is Brodenbach, which lies at the opening of a narrow ravine; this is full of mills, and makes an easy and picturesque walk for the most indifferent pedestrian. Only half an hour distant lies Ehrenburg, which some consider to be the finest ruin on the Moselle.

### TRAVEL NOTES AND QUERIES.

Rules in Regard to Correspondence for this Section.—All questioners must use a pseudonym for publication, but the communication must also bear the writer's own name and address as well, which will be regarded as confidential. All such communications to be addressed "Travel Editor, 'Nursing Mirror,' 28 Southampton Street, Strand." No charge will be made for inserting and answering questions in the inquiry column, and all will be answered in rotation as space permits. If an answer by letter is required, a stamped and addressed envelope must be enclosed, together with 2s. 6d., which fee will be devoted to the objects of the "Hospital" Convalescent Fund. Any inquiries reaching the office after Monday cannot be answered in "The Mirror" of the current week.

Venice in Summer (Marigold).—I think you can certainly remain till the end of June. May and June are ideal months in Venice Several hotels will take you at the price you mention. The Hotel Pension Bellevue and Hotel Milan, and Pension Anglaise. This last is very well situated. Still cheaper but well placed on the Riva degli Schiaroni is the Pension Kirsch. Gondolas are taken generally by the hour and are very cheap; for the first hour 1 lira, for each additional hour 50 centissimo. At night the tariff is dearer. The gondolier expects a trifle in addition. The ferries are always 5 centissimi till night when the charge is doubled.

In Old Provence (Artist).—It is useless to go to that region for so short a holiday. The journey is very expensive, and though living is cheap, if you do not mind roughing it a little, you would not under six weeks make up the loss of the journey. Moreover, in July, I think you would find it terribly hot in those enclosed valleys. You say you could alter your plans and so have longer time next year. If you can do that start not later than March or the 1st of April: you would then have the three best months before you for that country. I shall be pleased to advise you as to exact localities nearer the time. Living in the mountain towns is very rough, but I gather that you are prepared to face that state of things.

Glasgow Exhibition (L. W.).—We do not reply by letter exeept in accordance with the rules at the head of this column. I do not see that I can further enlarge upon the information given in the article of June 8th. What to see and what to leave unseen entirely depends on individual taste. My idea would be to get one of the numerous cheap guides to the show beforehand and then you can plan out your time to the best advantage. You would find the "Old Waverley," 135 Buchanan Street, fairly reasonable, but all are dear in Scotland.

## Figures and Tables

**Figure f1:**